# Contribution of model organism phenotypes to the computational identification of human disease genes

**DOI:** 10.1242/dmm.049441

**Published:** 2022-08-03

**Authors:** Sarah M. Alghamdi, Paul N. Schofield, Robert Hoehndorf

**Affiliations:** 1 Computational Bioscience Research Center, King Abdullah University of Science and Technology, 4700 KAUST, 23955 Thuwal, Saudi Arabia; 2 Department of Physiology, Development and Neuroscience, University of Cambridge, Downing Street, Cambridge CB2 3EG, UK

**Keywords:** Model organism, Phenotype, Disease gene discovery, Ontology, Semantic similarity, Machine learning

## Abstract

Computing phenotypic similarity helps identify new disease genes and diagnose rare diseases. Genotype–phenotype data from orthologous genes in model organisms can compensate for lack of human data and increase genome coverage. In the past decade, cross-species phenotype comparisons have proven valuble, and several ontologies have been developed for this purpose. The relative contribution of different model organisms to computational identification of disease-associated genes is not fully explored. We used phenotype ontologies to semantically relate phenotypes resulting from loss-of-function mutations in model organisms to disease-associated phenotypes in humans. Semantic machine learning methods were used to measure the contribution of different model organisms to the identification of known human gene–disease associations. We found that mouse genotype–phenotype data provided the most important dataset in the identification of human disease genes by semantic similarity and machine learning over phenotype ontologies. Other model organisms' data did not improve identification over that obtained using the mouse alone, and therefore did not contribute significantly to this task. Our work impacts on the development of integrated phenotype ontologies, as well as for the use of model organism phenotypes in human genetic variant interpretation.

This article has an associated First Person interview with the first author of the paper.

## Abstract (precis)

**Editor's choice:** We investigated the use of model organism phenotypes in the computational identification of disease genes, identifying several data biases and concluding that mouse model phenotypes contribute most to computational disease gene identification.

## INTRODUCTION

The discovery and building of models of human phenotypes in non-human animals has, over the past half-century, proven to be of substantial importance in improving our understanding of human disease and its underlying biology (Aitman et al., 2011; Wangler et al., 2017; Brown, 2021; Baldridge et al., 2021), and is providing insights that may be used to develop new therapeutic and diagnostic capabilities. The amount of data available that relate genetics, in particular genetic variation, to phenotypes associated with disease, is increasing rapidly. For example, the Monarch Initiative lists more than 2 million phenotypic associations over more than 100 species from dozens of public resources (Shefchek et al., 2020). By comparing the similarities between phenotypic profiles, these data can be used to help understand gene function and to identify the genotypic origins of phenotypic variation, which has wide applications in the discovery of the etiology of disease and the identification of candidate disease genes.

The challenge of relating phenotypes across different species is very significant. The ontologies and controlled vocabularies used to describe phenotypes are species specific and often structured in markedly different ways (Gkoutos et al., 2017). In order to compare phenotypic profiles between species, several different approaches have been developed to create an overarching phenotype ontology, allowing the integration of phenotype–genotype data from multiple species. This can then be used for measuring phenotypic similarity between an instance of one species, for example a human with a genetic disorder, and phenotypes annotated to multiple species and genotypes. This approach mobilizes the huge amount of genotype–phenotype data available in public databases such as Mouse Genome Informatics (MGI) (Eppig et al., 2017; Ringwald et al., 2021), FlyBase (Larkin et al., 2020) and Online Mendelian Inheritance in Man (OMIM) (Amberger et al., 2018), and maximizes the possibility of finding a phenotype annotation to a potential disease gene where such a relationship has not yet been reported in humans.

The development of a phenotype ontology covering both humans and model organisms has been essential to this task. The main approaches use evolutionary homology (and analogy) between anatomical structures (Mungall et al., 2012) and physiological processes, formalize these in a knowledge base or ontology, and infer relations between phenotypes using automated reasoning (Matentzoglu et al., 2019; Hoehndorf et al., 2011).

Loss-of-function phenotypes are available for several model organisms. These phenotypes have been generated through both hypothesis-driven experiments and large-scale reverse genetics experiments (Brown et al., 2018; Peterson and Murray, 2021). The genotype–phenotype data from model organisms have been used to discover human disease-associated genes using measures of phenotype similarity (Smedley and Robinson, 2015; Meehan et al., 2017; Hoehndorf et al., 2011). For this purpose, cross-species phenotype ontologies have been developed that systematically relate phenotypes of different organisms to each other (Gkoutos et al., 2017). The underlying assumption of phenotype-based methods to discover disease-associated genes is that genes function in evolutionarily conserved pathways or modules, and phenotypes associated with a loss or change of function in a gene are similar to phenotypes observed in a loss or change of function in the human ortholog of that gene (Oti and Brunner, 2007; McGary et al., 2010; Oti et al., 2008; Barabási et al., 2010). These methods are not only used to identify disease-associated genes but also to interpret and prioritize genomic variants associated with disease in tools that combine variant pathogenicity prediction with ranking of candidate genes (Cipriani et al., 2020; Boudellioua et al., 2017).

Phenotype-based methods to identify candidate genes associated with a set of phenotypes in humans are highly successful when the human gene has already been identified as a disease gene and is therefore associated with phenotypes (Köhler et al., 2009), and there are many examples where mouse phenotypes closely resemble human phenotypes and have therefore been used to identify disease-associated genes in humans (Meehan et al., 2017; Brommage et al., 2019; Smedley et al., 2021). Identification of candidate Mendelian disease genes using high-throughput screening suggests that this strategy might be able to identify candidates for inherited diseases of unknown genetic etiology. For example, out of 3328 genes screened in the mouse, potential models for 360 diseases were reported, including novel candidates (Meehan et al., 2017). More recently, the International Mouse Phenotyping Consortium (IMPC) reported knockouts of 1484 known disease genes, approximately half of which showed phenotypic similarity to human diseases using the Phenodigm platform (Cacheiro et al., 2019). It is estimated that, of the 16,847 mouse genes with a human ortholog, 79.9% have a null allele, either derived from hypothesis-driven experiments or large-scale screens such as the IMPC (Peterson and Murray, 2021); there are currently 3381 genes with mouse–human orthologs for which there are no corresponding mouse loss-of-function phenotypes. MGI reports 1694 human diseases with one or more mouse models and 7142 mouse genotypes modeling human diseases (MGI version 6.17; 14 December 2021), but their interpretation is complicated by the inclusion of dominant inheritance and multigenic or humanized models. It has been suggested that the ‘phenotype gap’ might be filled with genotype–phenotype associations from non-mammalian organisms with complementary coverage to the mouse and where loss-of-function mutations in mouse–human orthologs have no phenotype data (Mungall et al., 2016). To date, the contribution of different model organisms to the computational phenotype-driven identification of human disease genes has not been critically evaluated, an assessment that is important for the continued development of strategies and computational approaches to disease gene discovery. It is important to understand and quantify the contribution of more evolutionarily distant model organisms to discovering human disease-associated genes using the methods that have so successfully been applied to the mouse, in particular as, for example, zebrafish phenotypes are used in methods for disease gene discovery and human genetic variant interpretation (Wangler et al., 2017; Smedley et al., 2015, 2016).

Here, we used two different cross-species ontologies and several state-of-the-art methods for phenotype-based identification of disease-associated genes to evaluate the contribution of mouse, zebrafish, fruit fly and fission yeast loss-of-function phenotypes to discovering human disease genes. We found that only the mouse consistently predicts disease genes whereas the organisms that are more distant do not contribute. As part of our analysis, we found that our evaluation was affected by several biases in how orthologs of disease-associated genes are annotated in model organism databases, as well as how phenotype-based methods exploit these annotations; we analyzed and corrected for some of these biases to support future work in relating phenotype data to human disease.

## RESULTS

### Contribution of model organisms to disease gene discovery

We collected phenotypes associated with loss-of-function mutations in mouse, zebrafish, fruit fly and fission yeast from model organism databases. The phenotypes are described using different organism-specific phenotype ontologies, and we combined the phenotypes using the integrated phenotype ontologies uPheno (Shefchek et al., 2020) and our extension of the PhenomeNET (Pheno-e) ontology (Hoehndorf et al., 2011). Both phenotype ontologies combine the classes that represent phenotypes in different model organisms within a single ontology, thereby allowing us to exploit relations between the phenotypes and compare them. Pheno-e and uPheno also include human phenotypes from the Human Phenotype Ontology (HP) (Köhler et al., 2021), thereby allowing us to relate mutant model organism phenotypes to human disease-associated phenotypes.

We used the Pheno-e and uPheno ontologies and the phenotypes associated with loss-of-function mutations and human Mendelian diseases to test whether, and how much, different model organisms contribute to the phenotype-based computational discovery of disease-associated genes. For the purpose of evaluating the predictive performance, we used two datasets of gene–disease association: a ‘human’ dataset, which includes associations of human genes with Mendelian diseases reported in the OMIM (https://www.omim.org/) database, and a ‘mouse’ evaluation set, which consists of associations of mouse genes with human disease and represents mouse models of human disease in the MGI database (Ringwald et al., 2021). Then, we measured the semantic similarity between the phenotypes resulting from a gene's loss of function and human diseases (see Fig. S1). For each disease, we ranked all genes by their phenotypic similarity to the disease; we then determined at which rank we identified orthologs of known disease-associated genes.

**Fig. 1.**
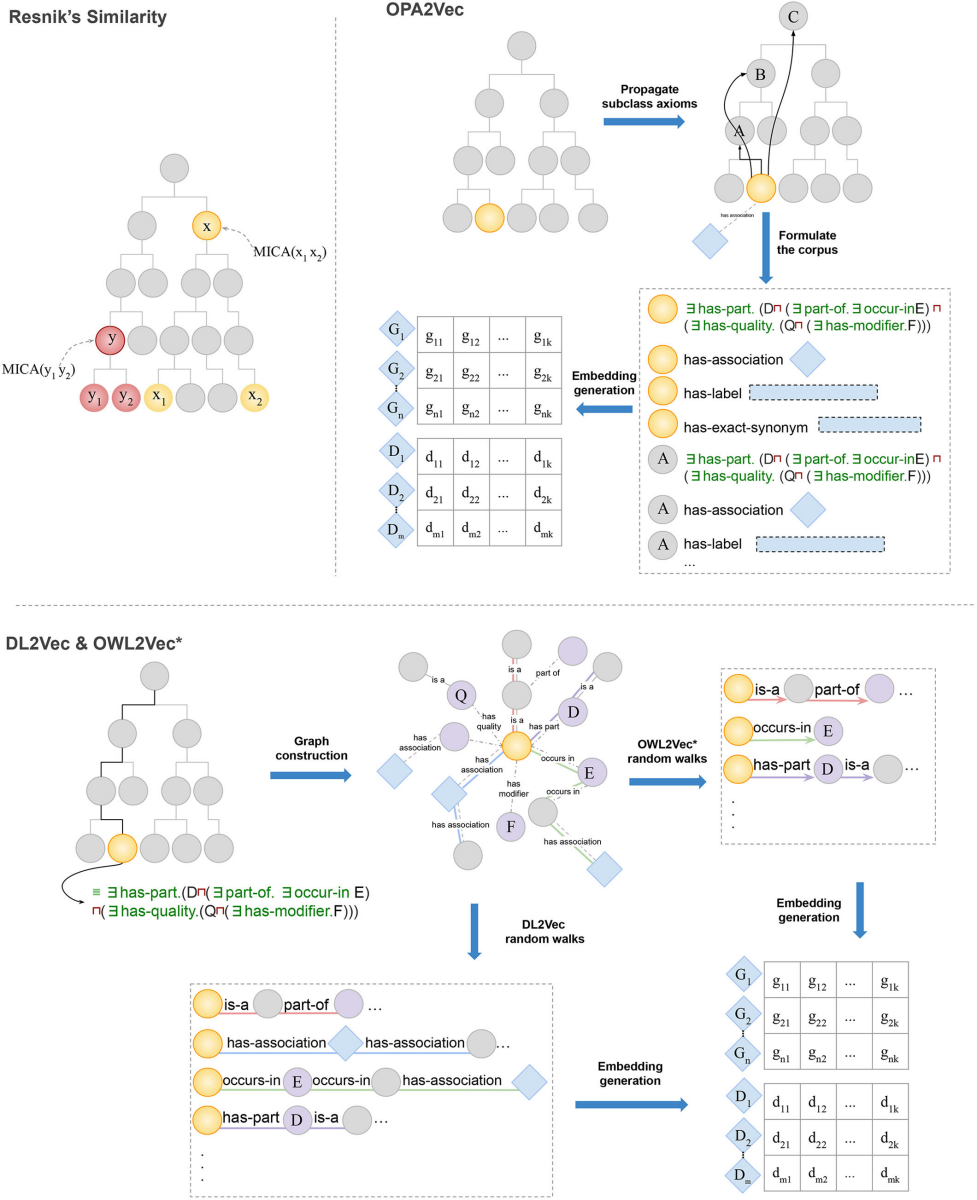
**Illustration of the approaches that we used to calculate phenotypic similarity.** Resnik's similarity uses the taxonomy of the ontology. OPA2Vec generates vector representations by using the axioms of the ontologies propagated over the subsumption hierarchy along with the natural language information available in the ontology. DL2Vec and OWL2Vec generate a graph from the ontologies axioms then perform random walks to generate vector representations for genes and diseases, with some differences including that the graphs are directed in OWL2Vec and undirected in DL2Vec.

This approach has repeatedly been successfully applied to discover disease-associated genes from model organisms through ontology-based computation of phenotype similarity (Meehan et al., 2017; Washington et al., 2009; Smedley et al., 2021) and further forms the foundation of several computational methods for finding disease-associated genomic variants (Smedley and Robinson, 2015; Smedley et al., 2016; Boudellioua et al., 2017). Multiple different approaches for determining phenotypic similarity have been developed, ranging from hand-crafted semantic similarity measures (Köhler et al., 2009; Smedley et al., 2013; Pesquita et al., 2009) to machine learning approaches (Smaili et al., 2018a; Chen et al., 2020). We used four different approaches to compute phenotype similarity between model organism phenotypes and human disease. First, we used Resnik's semantic similarity measure (Resnik, 1999), which relies on the taxonomic relations in the phenotype ontology to determine similarity between two sets of phenotypes. Resnik's similarity compares two phenotype classes; however, we needed to compare two sets of phenotype classes (i.e. all the phenotypes associated with the disease and all the phenotypes observed in the model organism). Consequently, we used the ‘best match average’ strategy (Pesquita et al., 2009) (see Materials and Methods) to combine multiple pairwise similarity measurements into a similarity between two sets of phenotypes. Resnik's similarity uses only the ontology taxonomy, whereas phenotype ontologies contain a large amount of additional information in the form of axioms that provide a computational description of the intended meaning of phenotype classes (Hoehndorf et al., 2015; Gkoutos et al., 2017). Therefore, we used the unsupervised machine learning method OPA2Vec (Smaili et al., 2018a), which is a deep learning method that learns a ‘representation’ of sets of phenotypes based on ontology axioms as well as natural language information contained in ontologies such as labels and definitions. As a third and fourth approach, we used the deep learning methods OWL2Vec* (Chen et al., 2021) and DL2Vec (Chen et al., 2020), which first converts ontology axioms into a graph, applies a random walk to explore the neighborhood of nodes in that graph and then generates a feature vector using Word2Vec. The aim of using these methods based on random walks was to exploit more ‘distant’ relations that arise through connecting multiple ontology classes. Fig. 1 illustrates the different approaches.

In our first experiment, we focused only on the groups of orthologous genes that have phenotype annotations in the mouse, zebrafish, fruit fly and fission yeast; the aim was to compare the contributions of different model organism to discovering gene–disease associations on the same set of associations from the ‘human’ dataset. There are 255 human genes with orthologous genes annotated with phenotypes in all organisms we considered, and, of these, 88 have a human ortholog associated with a Mendelian disease; several genes are associated with more than one Mendelian disease, and, in total, the 88 genes are associated with 173 Mendelian diseases.

We compared the phenotypic similarity of these genes to human disease phenotypes, and, within each organism, we ranked the genes by their similarity to each disease. We then evaluated the ranks at which we discovered the ‘correct’ gene (i.e. the gene with the human ortholog that is associated with the disease) and quantified the results using the area under the receiver operating characteristic (ROC) curve (ROCAUC) measure (see Materials and Methods). Table 1 summarizes the resulting performance. The results indicated that mouse mutant phenotypes can be used to reliably detect human disease-associated genes by all methods, whereas the other organisms do not consistently show a positive signal, and the quality of the signal is very dependent on the method used.

**Table 1.**
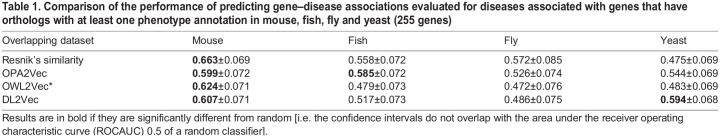
Comparison of the performance of predicting gene–disease associations evaluated for diseases associated with genes that have orthologs with at least one phenotype annotation in mouse, fish, fly and yeast (255 genes)

However, our observations were based on a relatively small set of 88 disease-associated genes that have orthologs with phenotypes in all organisms we studied. Therefore, we analyzed all genes with phenotypes in the different model organisms separately, incorporating genes that may lack phenotype annotations in other model organisms. We were able to test 11,672 human genes that have an ortholog in the mouse with phenotype annotations, 3418 genes in the fish, 6462 genes in the fruit fly and 1871 genes in yeast. As in our first experiment, we determined the phenotypic similarity using different semantic similarity measures and evaluated how well-established associations can be recovered.

Table 2 summarizes the ROCAUC values for each organism using the four approaches (Resnik’s similarity, OPA2Vec, OWL2Vec* and DL2Vec), and Tables S1 and S2 show the results for the Alliance dataset (Agapite et al., 2022). Similar to the first experiment, mouse phenotypes showed the highest performance across all methods we considered, and the mouse was the only organism for which all four methods to compute phenotype similarity showed a predictive performance that was better than random. Resnik’s similarity showed better-than-random performance for zebrafish and fruit fly phenotypes, but other methods predicted disease-associated genes no better than a random classifier (except DL2Vec in fission yeast using human gene–disease associations). In evaluations based on ontology embedding methods, the predicted performance was even significantly ‘worse than random’ (i.e. significantly below the ROCAUC 0.5 of a random classifier); this indicates that increased phenotypic dissimilarity between a gene and disease is associated with a higher chance of the gene and disease being associated, a rather counter-intuitive result that requires further exploration. We tested the hypothesis that these results are due to a study bias that results in an increased (phenotypic) distance due to the ontology structure. We broke this hypothesis into two parts; first, we hypothesized that genes that have an ortholog that is associated with a Mendelian disease in humans have more, and more specific, phenotype annotations than genes for which an ortholog is not associated with a Mendelian disease (or for which no human ortholog is known); this hypothesis tests for a form of study bias within the phenotype annotations. We found that disease-associated genes have a significantly higher total information content compared to non-disease associated genes (mouse, *P*=1.361×10^−43^; fish, *P*=6.793×10^−20^; fly, *P*=1.115×10^−12^; yeast, *P*=0.003; unpaired, one-tailed Student's *t*-test).

**Table 2.**
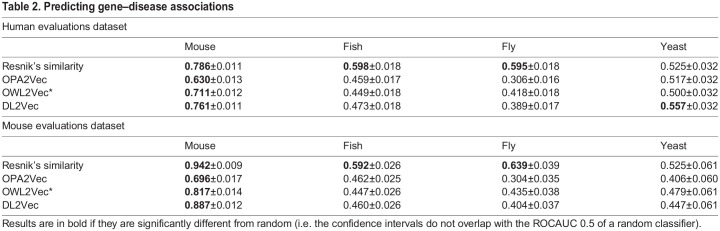
**Predicting** **gene–disease associations**

If these phenotypes do not match human disease-associated phenotypes well, the distance between these specific (i.e. ‘deep’ within the ontology hierarchy) phenotypes and general (i.e. ‘shallow’ within the ontology hierarchy) human phenotypes is higher than for less specific phenotypes; for example, the distance between the very general human phenotype class ‘phenotypic abnormality’ (HP:0000118) and the general fly phenotype ‘phenotypic abnormality of organism’ (FBbtAB:00000001) is less than the distance between ‘phenotypic abnormality of organism’ (FBbtAB:00000001) and the more specific class ‘phenotypic abnormality of eye dorsal compartment’ (FBbtAB:00111608). To further test whether this holds true across all genes with disease-associated and non-associated homologs in human, we calculated the absolute difference in information content between the phenotypes of the fly model and the most informative human phenotype superclass; the average difference in information content for genes with disease-associated human orthologs was 44, whereas the average difference in information content was 14 for genes with non-associated orthologs (*P*≤1.10×10^−60^, unpaired, one-tailed Student's *t*-test; see Supplementary Materials and Methods and Fig. S2). The only method that was not based on distances in our test is Resnik's similarity (which relies on the information content of the most informative shared ancestor), and this was also the only method not showing ROCAUCs below 0.5. Overall, these tests demonstrated that the ROCAUC results significantly lower than 0.5 are due to study bias combined with how the similarity methods utilize the ontology structure to determine similarity (i.e. based on distances traversed between classes).

As the mouse was the only model organism that consistently predicted gene–disease associations, we tested whether combining mouse phenotypes with other organism phenotypes would change the prediction results, i.e. whether combining information from multiple model organisms can improve predictions (i.e. test whether phenotypes of different organisms complement each other). We tested this on varying sets of genes depending on whether they have phenotypes in two model organisms. Table S3 shows the results. We found that combining mouse phenotypes with phenotypes of other model organisms did not significantly change the prediction results.

So far, we had performed our analysis only using the Pheno-e ontology. It was unclear whether our results demonstrated an inability of the Pheno-e ontology to compare phenotypes adequately or whether they reflected a property of the underlying data and the methods used to analyze it. Consequently, we used the cross-species phenotype ontology uPheno (Shefchek et al., 2020) and repeated the same analysis of predicting gene–disease associations using the four phenotype similarity computation methods; the results and comparison to Pheno-e are shown in Table 3. The results indicated that Pheno-e and uPheno have comparable performance and do not consistently show significant differences in predictive performance across different model organism and analysis methods.

**Table 3.**
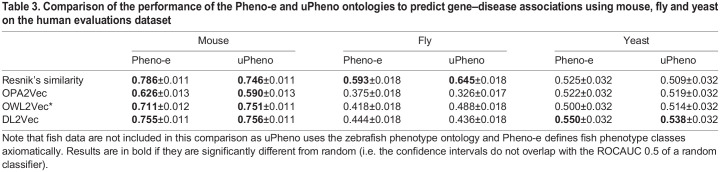
**Comparison of the performance of the** **Pheno-e and uPheno ontologies to predict gene–disease associations using mouse, fly and yeast on the human evaluations dataset**

We explored the characteristic scope of input data from different organisms, and how intrinsic bias in coverage of the genome–phenome space cognate with humans might affect the contribution of each organism to computational prediction. For example, some species lack organ systems present in humans, and others have quite distant physiology, for example in the immune system. There may also be biases in the selection of experimental systems between different model organisms, dependent partly on previously demonstrated value of those systems and on the historical development of study; furthermore, tractability, proven value for a particular area of investigation or cost may also explain differences between model organisms. Consequently, we investigated the predictive performance for different types of diseases separately, using the top-level classification of diseases in the Disease Ontology (DO) (Schriml et al., 2021) (see Tables S4-S51). Our analysis of the disease classes to which different model organisms contribute most showed that, for example, fly and fish contribute to diseases of the brain, central nervous system and nervous system. The fruit fly demonstrates substantial predictive performance for mental diseases and behavioral diseases, whereas yeast is predictive mainly for metabolic disorders.

### Supervised prediction

One advantage of similarity measures that rely on embeddings is that they can be used as input to ‘supervised’ machine learning approaches and thereby give rise to supervised similarity measures (Smaili et al., 2018b). In supervised machine learning, some examples of existing and absent associations between genes and diseases are used to train a model that can determine whether a new gene–disease pair is associated or not. Using the ontology embeddings as input to supervised machine learning methods has previously resulted in significantly improved prediction of gene–disease associations (Smaili et al., 2018a).

We trained a machine learning model (an artificial neural network), and used the output of this model to classify pairs of gene and disease embeddings into two classes, depending on whether the gene is associated with the disease (positives) or not (negatives). We evaluated the performance using a 10-fold cross-validation strategy (see Materials and Methods); Table 4 shows the results. We found that the supervised machine learning approach improved the predictive performance significantly over the unsupervised similarity-based approach, not only when using mouse model phenotypes but also for all other organisms. Furthermore, the supervised model was able to predict gene–disease associations significantly better than a random classifier using all embedding methods and organisms, and further improved significantly over all unsupervised prediction approaches.

**Table 4.**
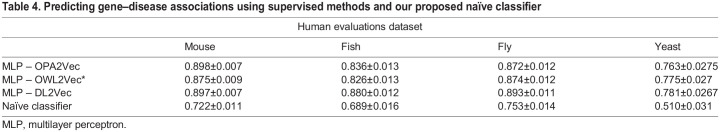
Predicting gene–disease associations using supervised methods and our proposed naïve classifier

However, although the predictive performance was substantially higher than random, it was somewhat surprising that the predictive performance when using phenotypes from distant organisms such as fly or fish matches the performance of using mouse phenotypes, and that even yeast phenotypes apparently are able to identify a large number of gene–disease associations quite accurately when there are so few orthologous genes [estimated to be around 2000 (O'Brien, 2004)], many without known disease associations in OMIM, the evaluation dataset. Neural networks may be able to exploit non-biological signals in training datasets to achieve relatively high predictive performance without producing biologically meaningful prediction results. For example, genes that are well studied and have a higher number of annotations may be associated with more diseases, or may more likely be associated with diseases; ranking genes higher solely based on the number of annotations they received could therefore improve prediction performance even without a specific biological signal. To test this hypothesis, we designed a ‘naïve’ classifier that predicts gene–disease associations solely based on the sum of the information content of phenotypes within a gene, i.e. it can be used to test whether genes that are annotated with more and more specific phenotypes are more likely associated with any disease. The naïve classifier ranks all genes based on the sum of the information content of their phenotype annotations, and predicts, for each disease *D*, the genes in descending order ranked by their information content; this prediction is independent of the disease *D*, i.e. the same list of genes is predicted in the same order for each disease (see Materials and Methods). The results of the prediction by the machine learning model, together with the naïve classifier results, are shown in Table 4. The results demonstrated that there is substantial bias in the underlying data that can be exploited by the naïve classifier, and is likely exploited by the machine learning models as well.

## DISCUSSION

We have evaluated the contribution of different model organism phenotypes to the computational identification of human gene–disease associations through the use of a variety of semantic similarity and machine learning methods. We find that the main contribution towards discovering human disease-associated genes using these methods comes from mouse phenotypes, whereas other model organism data do not contribute significantly to this task. The premise that pooling genotype–phenotype data from multiple organisms to enhance the phenotype-driven prediction of human disease genes, or interpret human genetic variants, is in principle sound, and has driven the development of multiple cross-species phenotype ontologies. The assumption has been that, as long as the knowledge contained in the ontology is ‘true’, then this should help bridge the ‘phenotype gap’, i.e. the human genes that have no phenotype associations in human but do have in model organisms. However, a critical evaluation of the main types of methods in use, machine learning and semantic similarity, indicates that the contribution of the non-mammalian model organism phenotypes to this task is computationally insignificant in comparison to mouse data. We identified two problems with the inconsistency of the results obtained by different methods: the first is bias generated by the use of the structure of the cross-species ontologies available, and the second is issues such as annotation density; however, there may be further biases that affect the results.

We tested the impact of a number of different parameters on our findings. First, our results hold true across two cross-species phenotype ontologies, Pheno-e and uPheno (Matentzoglu et al., 2019). Both ontologies have similar content and goals but are based on different ontology design patterns (Gkoutos et al., 2017; Alghamdi et al., 2019). We compared the two ontologies in our analyses to test whether the underlying ontology design patterns have a significant impact, but we did not consistently identify significant differences between both ontologies, indicating that our results hold true independent of the choice of phenotype ontology. Further, we used different analysis methods, focusing on traditional semantic similarity measures (Pesquita et al., 2009) that are largely defined based on explicit assumption of how similarity should be computed, as well as methods based on unsupervised and supervised machine learning with ontologies (Kulmanov et al., 2020). An increased number of, and more specific, annotations will bias estimates of semantic similarity. The effect of these biases has been demonstrated when comparing model organism and human disease phenotypes (Kulmanov and Hoehndorf, 2017), and also when predicting gene–disease associations where this bias can be corrected when detected (Cornish et al., 2018). The machine learning methods we employed are largely based on ‘paths’ in graph-based representations of the ontologies, whereas the semantic similarity we used is based on information content of classes without considering ‘paths’ explicitly; in particular, ‘distance’ is not a relevant consideration in our chosen semantic similarity measure, whereas distance is relevant in the machine learning methods we considered. We find that the notion of distance introduces a bias in prediction results, similar to biases found in some semantic similarity measures (Kulmanov and Hoehndorf, 2017; Cornish et al., 2018); using these methods should consequently be considered carefully, in particular as their black box nature makes it challenging to identify the reason for a prediction.

We identified and tested the impact of different biases within phenotype-based methods for finding candidate genes. We found a general study bias where disease-associated genes (or genes for which the human ortholog is disease associated) have generally more, and more specific, annotations than non-associated genes, and this affects not only semantic similarity measures but also machine learning methods; even more concerning, supervised machine learning methods can exploit biases in the data to make accurate predictions based on non-biological properties of the data (such as number and type of phenotype annotations). Again, use of black box models such as neural networks presents the danger of hiding the biases and how they are utilized in decision making.

We demonstrate here that assessment of the contribution of different model organisms to disease gene identification depends critically on the methods used, and we present evidence that supervised machine learning methods systematically overestimate the contribution of some model organisms, mainly by exploiting biases in phenotype data. Similar biases affect the evaluation of gene–disease and variant pathogenicity prediction methods (Grimm et al., 2015), and are challenging to detect and correct for. In the future, evaluation datasets and methods need to be developed that are less likely to overfit to biases in training and testing data, generalize well across different organisms and are robust to noise in phenotype annotations.

**Fig. 2.**
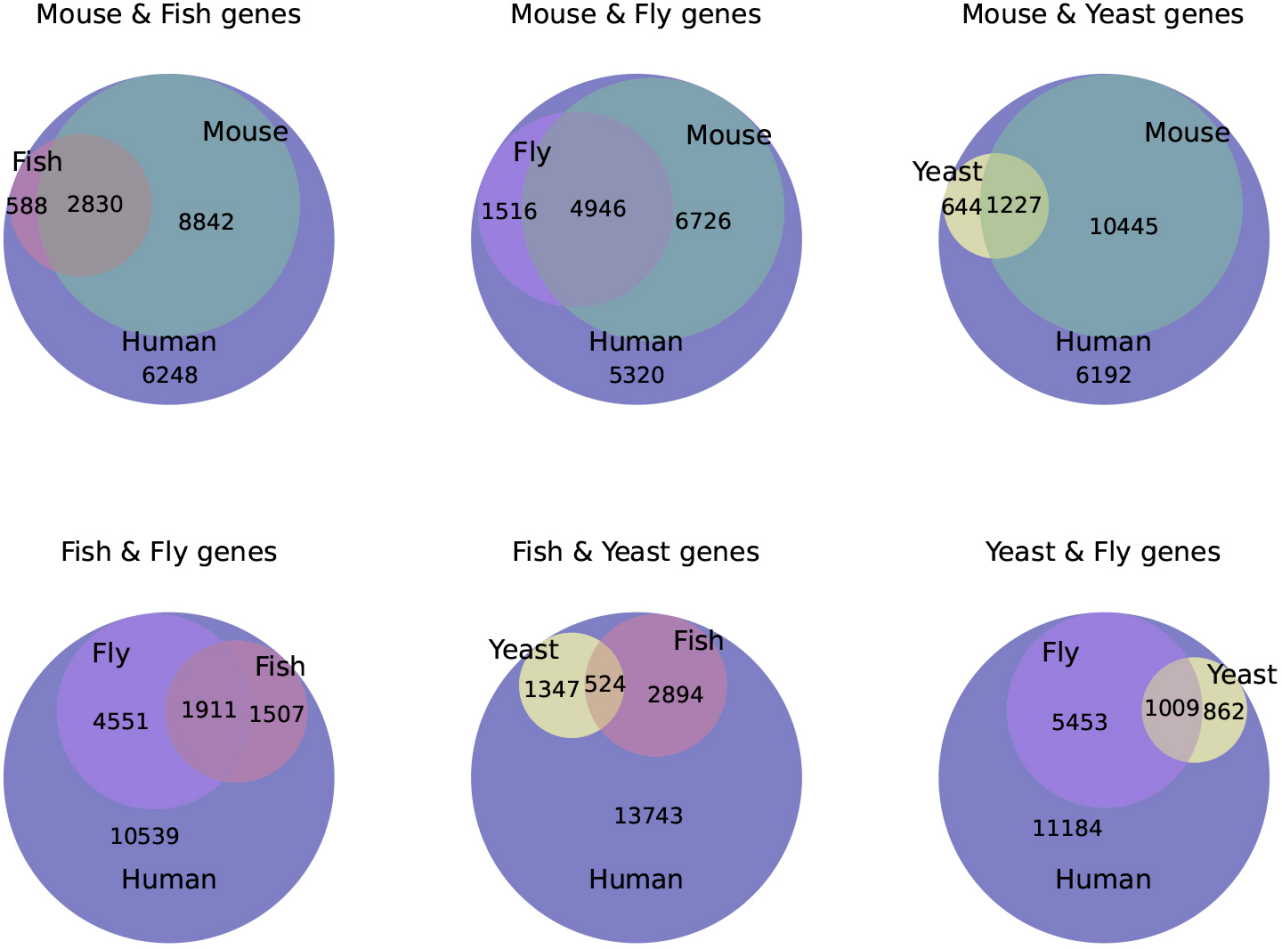
**Human genes with model organism orthologs.** The pairwise intersection of model organisms with phenotypes is illustrated in subgraphs. Each of these subgraphs represents 18,508 human genes in total.

The use of model organisms for understanding genotype–phenotype relations in humans is well established as a valuable strategy. Few organisms present a complete model of the human (Sundberg et al., 2013), but aspects of, for example, a disease phenotype might be studied more conveniently and with good fidelity in certain species or strains, yielding valuable insights from several model organisms (Hmeljak and Justice, 2019). Although the mouse is anatomically and physiologically closest to humans, and therefore phenotypes are more likely to be easily related, we know that, in particular systems or metabolic pathways, valuable information can come from much more distant organisms, for example insights into cell cycle control or aging from yeast (Pardo and Boriek, 2020). The computational use of model organism phenotypes to identify the underlying genetics of disease is a recent development, based on the premise that, as we do not have functional information for all genes in humans, combining this knowledge from model organisms can massively increase the knowledge that can be brought to bear (Mungall et al., 2010). Based on our data, there are 13,789 human genes that have an ortholog in the model organisms we investigated, which have been assigned one or more phenotypes (11,672 human genes have an ortholog in the mouse with phenotype annotations, 3418 genes in the fish, 6462 genes in the fruit fly and 1871 genes in yeast), and therefore over 63% of human genes have orthologs in model organisms with phenotype annotations (Willyard, 2018). Fig. 2 shows the pairwise overlap of genes with phenotypes in mouse, fish, fly and yeast.

A question not so far addressed is which of these annotations add to the power of computational approaches to discover disease-associated genes. By using two cross-species phenotype ontologies, we were able to show that the data from the mouse explain the majority of the human disease gene associations and that very little, if any, data from other model organisms contribute to this task. A previous focused study comparing mouse and fish phenotypes to predict disease genes in different disease categories for the Phenodigm algorithm (Oellrich et al., 2014) also showed the mouse to be overall more useful, but suggested that the zebrafish made contributions in specific disease areas. The authors suggested that this may be due to increased coverage of cardiovascular diseases in the data from mutant fish. Our findings are consistent with this, and also suggest that, as a consequence, the resulting performance is due to biases in the number and specificity of annotations and not due to the intrinsic relatedness between phenotypes.

We further analyzed the broader disease classes to which model organisms contribute (Tables S4-S51). Our results are consistent with the common selection of model organisms for different disease groups. For example, zebrafish are widely used in models of cardiovascular disease (Dahme et al., 2009; Prykhozhij and Berman, 2018; Narumanchi et al., 2021), and yeast is used as a model of metabolic disorders (Cervelli and Galli, 2021). Interestingly, we did not identify model organisms besides the mouse for immune system or eye disease including that of the retina. Our analyses reflect the intrinsic strengths of each model organism and the bias in the choice of organism to use when investigating particular types of disorders, either as a consequence of the utility of the models developed or the importance of the diseases modeled.

A deeper consideration of the differences and commonalities between phenotype–genotype relations in model organism species indicates why an analysis at a phenotypic level may be particularly sensitive to evolutionary distance. Intuitively, different organisms have different ontogeny and anatomy; for example, fish have fins that are homologous to mammalian limbs but differ in organization, and flies have wings and legs that have no homologs in mammals. Mutations in genes associated with phenotypes in fish and flies are often not associated with comparable phenotypes in humans simply because the structures are lacking or are profoundly different. For example, the human ortholog of the *dishevelled* gene (*dsh*) in *Drosophila*, which affects segment polarity, causes autosomal-dominant Robinow syndrome (OMIM #616331) (Patton and Afzal, 2002); yet, the phenotypes are not readily relatable because most of the structures affected in the human have no homologs in the fly. Nevertheless, decades of experimental investigation show that the underlying molecular processes in which these genes are involved are highly conserved but have evolved to be used in different morphogenetic or physiological processes (Wangler et al., 2017). Recognition of this problem has led to the concept of orthologous phenotypes or phenologs relating different phenotypes in different organisms resulting from mutation in the orthologous gene, but these are very difficult to identify in a phenotype-led approach (McGary et al., 2010). For similar reasons, it is challenging to predict phenotypic pleiotropy in genetically distant organisms due to the inevitable differences in genome, genetic interactions, anatomy and physiology (Wagner and Zhang, 2011; Chesmore et al., 2017), and the fact that phenotypes are emergent properties of an organism (Varela et al., 1974). This means that model organisms can be extremely valuable in understanding and investigating the molecular mechanisms underlying normal physiology as well as pathophysiology, but identifying the participants in such processes from whole-organism, or often even cellular, phenotypes can be extremely difficult and species dependent (Kulmanov and Hoehndorf, 2020). Conversely, we have examples where careful phenotypic characterization of mouse models has identified the genetic origin of human diseases and in some cases expanded the phenotypic characterization of quite familiar disorders (Brommage et al., 2019; Thiele et al., 2012), emphasizing the power of phenotypic homology in closely related organisms.

Our study illustrates some of the limitations in the utility of non-mammalian phenotype–genotype data in computational discovery of genes responsible for human disease. It highlights more broadly intrinsic problems in ‘black box’ methods for machine learning, the weaknesses of phenotype ontologies and methods for estimating semantic similarity, data overfitting and the intrinsic biases in data collection. We hope that understanding these problems will help in the development of new approaches, possibly new ontological tools, and inform the collection and annotation of new data.

## MATERIALS AND METHODS

### Ontologies

Several foundational ontologies were used for the axiomatization of species-specific phenotype ontologies, and we reused them for the construction of the Pheno-e ontology. We used the following ontologies: Gene Ontology (GO) (Ashburner et al., 2000), downloaded from http://purl.obolibrary.org/obo/go.owl; Cell Ontology (CL) (Diehl et al., 2016), downloaded from http://purl.obolibrary.org/obo/cl-basic.owl; Phenotype and Trait Ontology (PATO) (Gkoutos et al., 2005), downloaded from http://purl.obolibrary.org/obo/pato.owl; Uber Anatomy Ontology (UBERON) (Mungall et al., 2012), downloaded from http://purl.obolibrary.org/obo/uberon.owl; Zebrafish Anatomy and Development Ontology (ZFA) (Van Slyke et al., 2014), downloaded from http://purl.obolibrary.org/obo/zfa.owl; Neuro Behavior Ontology (NBO) (Gkoutos et al., 2012), downloaded from http://purl.obolibrary.org/obo/nbo.owl; Biological Spatial Ontology (BSPO) (Dahdul et al., 2014), downloaded from http://purl.obolibrary.org/obo/bspo.owl; and *Drosophila* Gross Anatomy Ontology (FB-BT) (Costa et al., 2013), downloaded from http://purl.obolibrary.org/obo/fbbt.owl.

The phenotype ontologies used were as follows: Mammalian Phenotype Ontology (MP) (Smith and Eppig, 2012), downloaded from http://purl.obolibrary.org/obo/mp.owl; Human Phenotype Ontology (HP) (Köhler et al., 2018), downloaded from http://purl.obolibrary.org/obo/hp.owl; *Drosophila* Phenotype Ontology (DPO) (Osumi-Sutherland et al., 2013), downloaded from http://purl.obolibrary.org/obo/dpo.owl; and Fission Yeast Phenotype Ontology (FYPO) (Harris et al., 2013), downloaded from http://purl.obolibrary.org/obo/fypo.owl. The latest version of the ontologies is used for every update of Pheno-e; the results reported here used ontologies downloaded in February 2021.

### Datasets and phenotype annotations

For constructing the model organism phenotype classes we used the following: (1) from the ontologies MP (Smith and Eppig, 2012), HP (Köhler et al., 2018), DPO (Osumi-Sutherland et al., 2013) and FYPO (Harris et al., 2013), we reconstructed the phenotype classes for mouse, human, fly and yeast, respectively; (2) from FlyBase (Thurmond et al., 2018), we used allele_phenotypic_data_fb_2021_01.tsv, which provides the alleles phenotypes association using controlled vocabulary for *Drosophila melanogaster*, and used this file to create the abnormal anatomy classes (FBabAB); (3) from ZFIN (https://zfin.org/), we used phenoGeneCleanData_fish.txt, which contains zebrafish gene–phenotype associations to create classes representing zebrafish phenotypes.

For generating representations of genes and diseases and for predicting gene–disease associations, we used the following files downloaded on 07 February 2021: (1) human disease–phenotype annotations were obtained from the HP database (Köhler et al., 2018) phenotype_annotation.tab, comprising manual and semi-automated annotations representing disease identifiers from the OMIM (Amberger and Hamosh, 2017), Orphanet (Weinreich et al., 2008) and DECIPHER (Firth et al., 2009) databases; (2) mouse gene–phenotype annotations were obtained from the MGI database (Ringwald et al., 2021) MGI_GenePheno.rpt, which uses MP (Smith and Eppig, 2012); (3) fly gene–phenotype annotations were generated using files from FlyBase (Thurmond et al., 2018), allele_phenotypic_data_fb_2021_01.tsv, which represents the allele–phenotype annotations, and fbal_to_fbgn_fb_2021_01.tsv, which contains allele–gene mappings; and (4) yeast gene–phenotype annotations were obtained from PomBase (Harris et al., 2013), phenotype_annotations.pombase.phaf.gz, which represent the phenotypes annotations for fission yeast *Schizosaccharomyces pombe*.

For evaluation purposes, we used two gene–disease association datasets from MGI (Ringwald et al., 2021). The first dataset is a ‘human’ dataset that includes associations of human genes with Mendelian diseases using identifiers from the OMIM database. MGI acquire this dataset for human gene–disease associations from OMIM and additional data from other sources including NCBI's Gene Review (Eppig et al., 2017). This dataset includes 2848 human gene, 3644 OMIM disease and 11,778 human gene–disease associations. The second dataset is a ‘mouse’ evaluation set that includes associations of mouse genes with human disease and represents mouse models of human disease in the MGI database. This dataset contains 2459 mouse genes, 2157 disease and 8101 mouse gene–disease associations. This dataset is acquired by curating data on mouse models from the scientific literature and high throughput experiments (Eppig et al., 2017). Both datasets are included in the file MGI_DO.rpt available from MGI. Gene–phenotype and gene–disease annotations are derived by manual curation (Bello and Eppig, 2016). The version we used was downloaded in February 2021. Additionally, we utilized data on gene–disease associations from the Alliance of Genome Resources (Alliance of Genome Resources Consortium*,* 2020), an effort to integrate data resources among the major model organism databases. We mapped DO (Schriml et al., 2022) identifiers to OMIM using the DO and MGI database cross references. As a result, we identified 4022, 2366, 2681 and 406 OMIM diseases that were associated with mouse, fish, fly and yeast genes, respectively.

To find orthologous genes between different organisms, we used several files. Human–mouse orthology was obtained from HMD_HumanPhenotype.rpt from MGI. Human–zebrafish and mouse–zebrafish orthologs were obtained from human_orthos.txt and mouse_orthos.txt from ZFIN. We obtained human–fly orthology from dmel_human_orthologs_disease_fb_2020_06.tsv from FlyBase. Human–yeast orthologs were obtained from https://www.pombase.org/data/orthologs/. We obtained mouse–fly and mouse–yeast orthologs from OMA (Train et al., 2017).

### Pheno-e and integration of model organism phenotypes

The Pheno-e was developed by utilizing existing phenotype ontology class descriptions and reformulating them according to a set of ontology design patterns so that different phenotype ontologies can be integrated (Hoehndorf et al., 2011). The uPheno ontology similarly establishes bridging axioms to connect phenotypes from different species-specific ontologies (Matentzoglu et al., 2019). The current version of the Pheno-e does not contain classes for yeast and fly phenotypes, whereas these two species are covered in the uPheno. We therefore expanded PhenomeNET to include phenotypes from fly and yeast. We obtained the phenotype class descriptions from the DPO and FYPO ontologies, and reformulated them using the PhenomeNET design patterns. The new classes we created use the pattern:
(1)

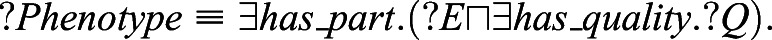
In this pattern, ?*E* characterizes the entity underlying the phenotype (either from an anatomy ontology or the GO) and ?*Q* is a quality from the PATO ontology. We used relations from the OBO Relation Ontology (Smith et al., 2005); the relations we used in constructing PhenomeNET and Pheno-e include ‘part-of’, ‘results-from’, ‘during’, ‘has-quality’, ‘has-central-participant’, ‘occurs-in’ and ‘towards’.

FlyBase has two types of abnormal phenotype classes; it associates alleles with classes from the DPO as well as with classes from the fly anatomy ontology, indicating that an anatomical or developmental structure was found to be abnormal in a mutant fly. In order to integrate those anatomical abnormalities in the Pheno-e and therefore use them in cross-species phenotype analysis, we added the abnormal anatomical structures as new classes in PhenomeNET and associated the alleles with these classes:
(2)

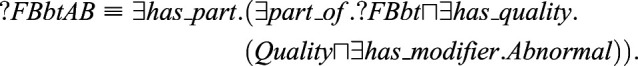
*Quality* and *Abnormal* are classes from the PATO ontology. For example, FBal0148512 is an allele associated with wing abnormalities (Végh and Basler, 2003), and we associated the allele with the newly defined class ‘phenotypic abnormality of wing’, defined accordingly to the pattern in Eqn 2, where ?*FBbt* is the class ‘wing’ from the fly anatomy ontology.

Similarly to PhenomeNET, we defined homologous and analogous anatomical structures as equivalent (for the purpose of the ontology). For example, we defined the nervous system in fly (FBbt:00005093) to be equivalent to the nervous system in the zebrafish (ZFA:0000396) and the nervous system in the Uberon multi-species anatomy ontology (UBERON:0001016). Through these equivalence class assertions, we deductively inferred an equivalence between ‘nervous system phenotype’ (MP:0003631), ‘abnormal neuroanatomy’ (FBcv:0000435) and ‘abnormality of the nervous system’ (HP:0000707), thereby enabling the direct comparison of mouse, fly and human phenotypes.

The extended Pheno-e contains 16,083 HP classes, 13,698 MP classes, 35,954 zebrafish phenotype classes (PHENO classes, defined in Pheno-e), 3111 fly phenotype classes (FBcv classes and abnormal anatomy FBbtAB classes) and 7636 yeast phenotype (FYPO) classes.

We used phenotype datasets consisting of 8031 OMIM diseases annotated with HP classes, 14,210 mouse genes annotated with MP classes, 6182 zebrafish genes annotated with PHENO classes, 13,512 fly genes annotated with FBbtAB and FBcv classes, and 4443 yeast genes annotated with FYPO classes.

Using automated reasoning over the Pheno-e ontology, we are able to infer relations between classes from different organisms; in particular, we are able to automatically infer whether two classes are equivalent or whether one class is a subclass of another class. We show the number of inferred relations in Pheno-e between the different species in Table 5, and for uPheno in Table 6. The tables show that it is possible to relate a large number of model organism phenotypes to human phenotypes through the Pheno-e and uPheno ontologies.

**Table 5.**
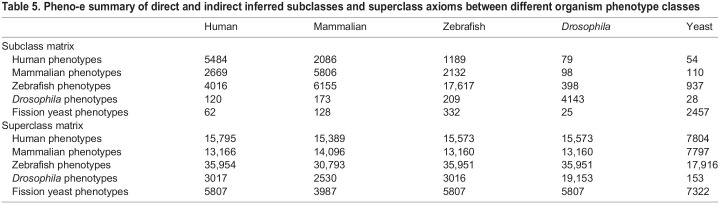
Pheno-e summary of direct and indirect inferred subclasses and superclass axioms between different organism phenotype classes

**Table 6.**
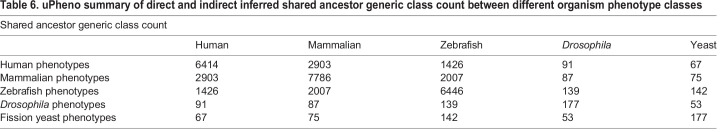
uPheno summary of direct and indirect inferred shared ancestor generic class count between different organism phenotype classes

**Fig. 3.**
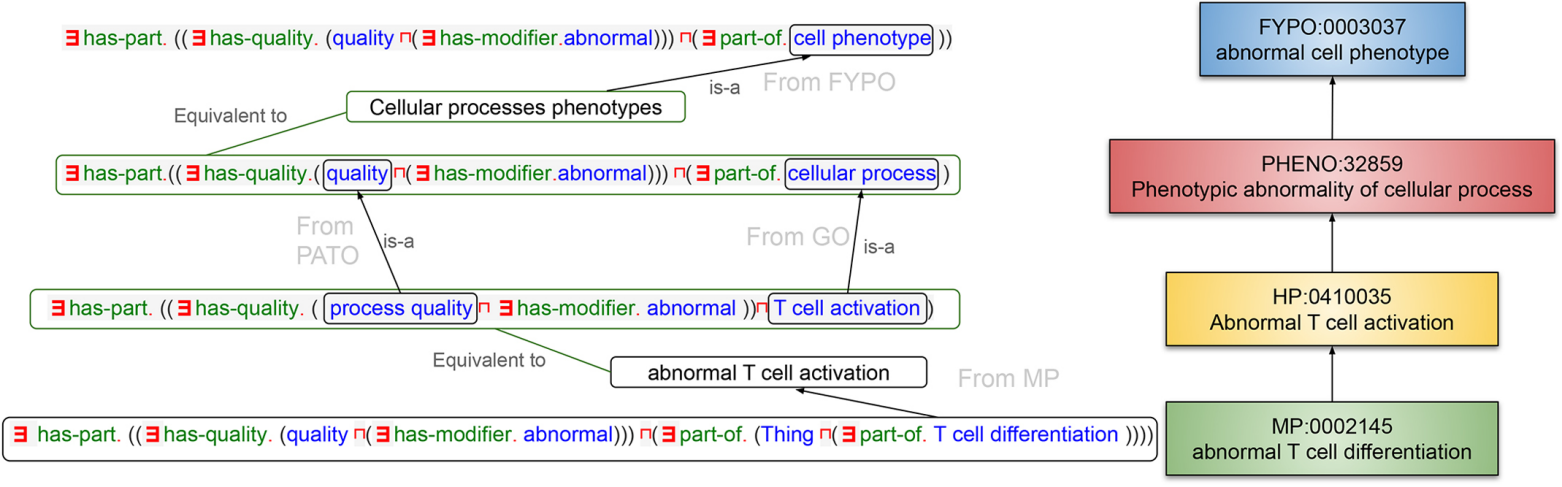
**Example of inferred hierarchy relating classes from different organism phenotypes.** The classes are colour coded according to the source from which they were obtained.

Fig. 3 illustrates an example of inferred relations between phenotype classes of different organisms and resources. In this example, the class ‘abnormal T cell activation’ (HP:0410035) has as a (zebrafish) superclass ‘phenotypic abnormality of cellular process’ (PHENO:32859). This inference was made because of the background available from PATO and GO, as the class ‘process quality’ (PATO:0001236) is a subclass of ‘quality’ (PATO:0000001), and ‘T cell activation’ (GO:0042110) is a subclass of ‘cellular process’ (GO:0009987).

### Phenotype similarity

We applied a set of different methods to compare the similarity of phenotypes associated with a loss-of-function model organism mutant and human disease phenotypes.

#### Resnik’s semantic similarity

We calculated Resnik’s similarity (Resnik, 1995) between genes and diseases annotated with phenotype classes; the use of integrated phenotype ontologies enabled the direct comparison of phenotypes. Resnik's similarity is a similarity measure based on information content, defined as:
(3)

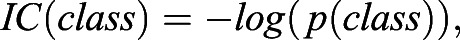
where the probability of a class is defined as the frequency of annotation with the class. The similarity between two ontology classes is defined as the information content of the most informative common ancestor (MICA) of two classes:
(4)

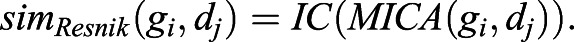
As we compared groups of classes, we used the best match average method (BMA) (Pesquita et al., 2009) to calculate the similarity between genes and diseases:
(5)

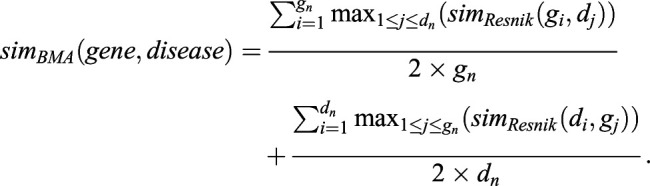


#### OPA2Vec

As second method, we ‘embedded’ phenotypes in a real-valued vector space using OPA2Vec (Smaili et al., 2018a); an embedding is a structure-preserving map from one algebraic structure (ontology axioms) into another (vector space), i.e. an embedding preserves (some) properties of the first structure within the second. OPA2Vec is mainly based on preserving syntactic relations in asserted and inferred ontology axioms.

We generated embeddings for diseases and genes using OPA2Vec based on the phenotypes associated with the diseases and genes and the axioms in the phenotype ontology. For the training, we used the skip gram, and set mincount to 0, embedding size to 100 and window size to 5. We then computed similarity between genes and diseases based on the cosine similarity of their embeddings.

#### DL2Vec and OWL2Vec*

Another method we used to generate feature embeddings is the DL2Vec (Chen et al., 2020) method. DL2Vec converts description logic axioms into undirected graph representations and uses a random walk to explore the graph; the walks are then treated as sentences and encoded using a language model. The graph is generated from the ontology axioms, and each phenotype class becomes one node in this graph; we added the gene and disease identifiers to this graph and connected them to the phenotype classes with which they are annotated. To generate the walks, we chose the walk length to be 30, with 50 number of walks, and we used a skip gram method with window size set to 10, mincount to 1 and embedding size to 100.

OWL2Vec* (Chen et al., 2021) is an embedding method similar to DL2Vec and based on a similar graph representation. OWL2Vec* graphs are directed and do not include equivalence or disjoint class axioms. We used random walker with walk depth 7 and 30 iterations with projection on structure document, and we used a skip gram method with window size set to 5, mincount to 1, negatives to 5 and embedding size to 100. For both DL2Vec and OWL2Vec* embeddings, we compared the phenotypic similarity between genes and diseases using cosine similarity.

### Prediction of gene–disease associations

In addition to predicting gene–disease associations based on phenotypic similarity, we also used supervised prediction of these associations. For this purpose, we used a multilayer perceptron (MLP) with a single hidden layer. The input of the MLP is the concatenated embeddings of a disease and a gene. We used a hidden layer half the size of the input and a binary output using a sigmoid function, indicating whether the gene and disease are associated through a gene–disease relation or not; we further used the value of the sigmoid to rank genes for a disease. We randomly generated five negatives to each positive. For the training, we used the Adam optimizer (Kingma and Ba, 2014 preprint) with a learning rate of 0.001 and maximum number of iterations of 300. To evaluate, we used 10-fold cross validation, stratified by diseases.

### Naïve classifier

We hypothesized that some of our results are due to imbalanced or biased data. To test this hypothesis, we defined a ‘naïve’ classifier that predicts gene-disease associations on the basis of the information content of phenotypes of a gene alone; this ‘classifier’ ranks all genes based on the sum of the information content of their phenotype annotations, sorts genes in descending order, and predicts the same ranked list of genes for each disease (i.e. the classifier is independent of the disease). The aim of this ‘naïve’ classifier was to test whether genes annotated with more and more specific phenotypes are generally more likely to be associated with a disease, i.e. it tests for a kind of annotation bias.

### Evaluating predictive performance

Our evaluation was based on estimating how well the different approaches rank disease-associated genes given a set of disease-associated phenotypes, for phenotypes from different organisms. Higher phenotypic similarity between a gene and a disease indicates higher likelihood that the gene (or its human ortholog) is associated with that disease. We evaluated two datasets from the MGI file MGI_DO.rpt, one for human gene–disease associations from OMIM and another MGI-curated dataset of mouse models of human disease.

For the evaluation, for each disease *D_i_* in our evaluation set, we ranked all genes *G_1_*,…,*G_n_* based on their phenotypic similarity to *D_i_*. For each disease *D_i_*, we determined the rank (or ranks) at which the associated gene (or genes) appears in this ranked list. We used this information to determine the false-positive and true-positive rate at each rank; we averaged the true- and false-positive rates across all diseases and used this to determine the ROC curve and the ROCAUC.

When using supervised methods to predict gene–disease associations, we used the same evaluation in a 10-fold cross-validation setting, and we ranked genes based on the output of the sigmoid unit of our machine learning model.

### Implementation

We used several tools and libraries, such as the OWLAPI (http://owlapi.sourceforge.net/) for generating the ontology groovy and Python scripts for data processing. We also used several Vython libraries, such as sklearn, numpy, pandas and PyTorch (Paszke et al., 2017) for the supervised learning. For calculating Resnik’s semantic similarity, we used the Semantic Measures Library (SML) (Harispe et al., 2013).

## Supplementary Material

Supplementary information
