# Supplementary material for: Contribution of model organism phenotypes to the computational identification of human disease genes
Source: Dis Model Mech. 2022 Aug 3;15(7):dmm049441. doi: 10.1242/dmm.049441 (PMC9366895; doi:10.1242/dmm.049441)
Supplement: Supplementary information [file dmm-15-049441-s1.pdf]

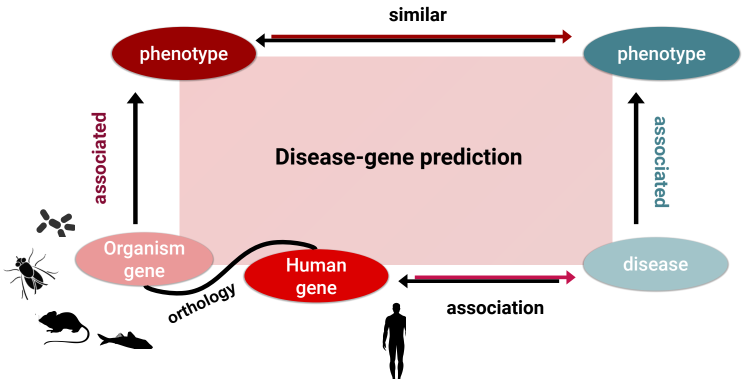

Fig. S1. Overview of gene–disease association prediction.

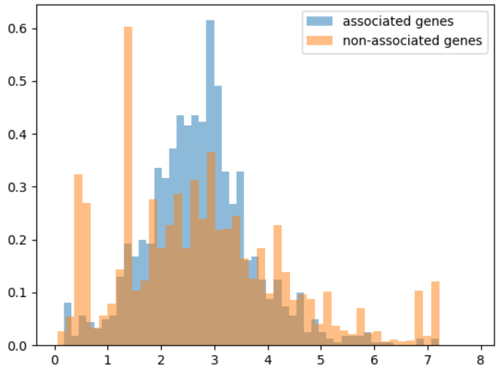

Fig. S2. Average information content difference between *Drosophila* gene phenotype annotations and m informative common human phenotype class in disease associated genes and non-disease associated genes.

Table S1. Evaluating the use of model organism phenotypes in the prediction of gene–disease associa- tions using the Alliance dataset with all the available associations.

	Mouse	Fish	Fly	Yeast
Resnik's	**0.753** ± 0.006	**0.575** ± 0.011	**0.601** ± 0.01	0.491 ± 0.041
OPA2Vec	**0.519** ± 0.007	0.359 ± 0.010	0.312 ± 0.009	0.420 ± 0.040
DL2Vec	**0.678** ± 0.007	0.464 ± 0.011	0.406 ± 0.010	0.477 ± 0.041
OWL2Vec*	**0.610** ± 0.007	0.445 ± 0.011	0.419 ± 0.010	0.470 ± 0.041
genes with phenotypes	14210	6182	11061	4443
associated diseases (mapped to OMIM)	4022	2366	2681	406
disease-gene associations	18017	8811	9840	595

Table S2. Evaluating the use of model organism phenotypes in the prediction of gene–disease associa- tions using the Alliance dataset, filtering out the automated assertions, and associations of genotype based on phenotypes and sequence alignment and similarity evidence.

	Mouse	Fish	Fly	Yeast
Resnik's	**0.933** ± 0.009	**0.691** ± 0.044	**0.621** ± 0.043	0.458 ± 0.062
OPA2Vec	**0.688** ± 0.017	0.262 ± 0.042	0.321 ± 0.04	0.465 ± 0.062
DL2Vec	**0.876** ± 0.012	0.440 ± 0.047	0.397 ± 0.042	0.478 ± 0.062
OWL2Vec*	**0.807** ± 0.014	0.385 ± 0.046	0.442 ± 0.045	0.492 ± 0.062
genes with phenotypes	14210	6182	11061	4443
associated diseases (mapped to OMIM)	1544	265	287	186
disease-gene associations	3057	445	498	256

Table S3. Comparison of performance in predicting gene–disease associations evaluated on diseases associated with genes which have orthologs with at least one phenotype: (a) in mouse and fish (2830 genes); (b) mouse and fly (4946 genes); (c) mouse and yeast (1227 genes), on the human evaluation data set

(a)	Combined	Fish	Mouse	#genes
**Resnik's**	0.488 ± 0.020	**0.531** ± 0.019	**0.793** ± 0.016	
**OPA2Vec**	**0.720** ± 0.018	0.464 ± 0.019	**0.691** ± 0.018	
**OWL2Vec**	**0.762** ± 0.017	0.487 ± 0.019	**0.721** ± 0.018	
**DL2Vec**	**0.778** ± 0.017	0.519 ± 0.019	**0.786** ± 0.016	2830

(b)	Combined	Fly	Mouse	#genes
**Resnik's**	0.470 ± 0.018	**0.555** ± 0.019	**0.767** ± 0.015	
**OPA2Vec**	**0.671** ± 0.017	0.444 ± 0.018	**0.656** ± 0.017	
**OWL2Vec**	**0.726** ± 0.016	0.492 ± 0.018	**0.693** ± 0.016	
**DL2Vec**	**0.739** ± 0.015	0.454 ± 0.018	**0.751** ± 0.015	4946

(c)	Combined	Yeast	Mouse	#genes
**Resnik's**	0.458 ± 0.037	0.458 ± 0.035	**0.655** ± 0.035	
**OPA2Vec**	**0.605** ± 0.036	**0.536** ± 0.035	**0.562** ± 0.037	
**OWL2Vec**	**0.625** ± 0.036	0.534 ± 0.035	**0.558** ± 0.037	
**DL2Vec**	**0.669** ± 0.035	**0.554** ± 0.035	**0.641** ± 0.036	1227

Table S4. Prediction of gene–disease association for diseases mapped to *nervous system disease* (DOID:863) – Human dataset

Method	mouse	fish	fly	yeast
Resnik's	**0.817 ± 0.024**	**0.593 ± 0.042**	**0.633 ± 0.042**	0.483 ± 0.083
OPA2Vec	**0.645 ± 0.030**	0.347 ± 0.041	0.348 ± 0.039	0.495 ± 0.083
DL2Vec	**0.767 ± 0.026**	0.430 ± 0.042	0.410 ± 0.040	0.563 ± 0.083
OWL2Vec*	**0.720 ± 0.028**	0.446 ± 0.042	0.418 ± 0.041	0.542 ± 0.083

Table S5. Prediction of gene–disease association for diseases mapped to *nervous system disease* (DOID:863) - Mouse dataset

Method	mouse	fish	fly	yeast
Resnik's	**0.955 ± 0.023**	**0.633 ± 0.077**	**0.670 ± 0.098**	0.434 ± 0.159
OPA2Vec	**0.755 ± 0.048**	0.337 ± 0.076	0.352 ± 0.093	0.451 ± 0.159
DL2Vec	**0.910 ± 0.032**	0.422 ± 0.079	0.454 ± 0.097	0.487 ± 0.160
OWL2Vec*	**0.835 ± 0.041**	0.432 ± 0.079	0.455 ± 0.102	0.437 ± 0.159

Table S6. Prediction of gene–disease association for diseases mapped to *disease of metabolism* (DOID:0014667) - Human dataset

Method	mouse	fish	fly	yeast
Resnik's	**0.719 ± 0.042**	0.546 ± 0.083	0.514 ± 0.067	0.425 ± 0.074
OPA2Vec	**0.647 ± 0.044**	0.384 ± 0.081	0.389 ± 0.061	**0.579 ± 0.074**
DL2Vec	**0.759 ± 0.040**	0.473 ± 0.083	0.486 ± 0.063	**0.579 ± 0.074**
OWL2Vec*	**0.714 ± 0.042**	0.444 ± 0.083	0.497 ± 0.064	0.499 ± 0.075

Table S7. Prediction of gene–disease association for diseases mapped to *disease of metabolism* (DOID:0014667) - Mouse dataset

Method	mouse	fish	fly	yeast
Resnik's	**0.924 ± 0.036**	0.572 ± 0.112	0.478 ± 0.136	0.416 ± 0.154
OPA2Vec	**0.677 ± 0.063**	0.366 ± 0.109	0.380 ± 0.124	0.591 ± 0.154
DL2Vec	**0.911 ± 0.039**	0.446 ± 0.113	0.488 ± 0.128	0.528 ± 0.156
OWL2Vec*	**0.814 ± 0.053**	0.451 ± 0.113	0.555 ± 0.134	0.538 ± 0.156

Table S8. Prediction of gene–disease association for diseases mapped to *inherited metabolic disorder* (DOID:655) - Human dataset

Method	mouse	fish	fly	yeast
Resnik's	**0.715 ± 0.042**	0.545 ± 0.086	0.512 ± 0.067	0.425 ± 0.074
OPA2Vec	**0.644 ± 0.045**	0.387 ± 0.084	0.394 ± 0.062	**0.579 ± 0.074**
DL2Vec	**0.756 ± 0.040**	0.472 ± 0.086	0.488 ± 0.063	**0.579 ± 0.074**
OWL2Vec*	**0.712 ± 0.043**	0.445 ± 0.086	0.497 ± 0.064	0.499 ± 0.075

Table S9. Prediction of gene–disease association for diseases mapped to *inherited metabolic disorder* (DOID:655) - Mouse dataset

Method	mouse	fish	fly	yeast
Resnik's	**0.918 ± 0.040**	0.570 ± 0.125	0.472 ± 0.138	0.404 ± 0.155
OPA2Vec	**0.690 ± 0.068**	0.372 ± 0.122	0.386 ± 0.128	0.604 ± 0.155
DL2Vec	**0.909 ± 0.042**	0.449 ± 0.125	0.502 ± 0.131	0.538 ± 0.158
OWL2Vec*	**0.838 ± 0.054**	0.454 ± 0.125	0.562 ± 0.135	0.548 ± 0.157

Table S10. Prediction of gene–disease association for diseases mapped to *central nervous system disease* (DOID:331) - Human dataset

Method	mouse	fish	fly	yeast
Resnik's	**0.775 ± 0.039**	**0.632 ± 0.064**	**0.644 ± 0.059**	0.468 ± 0.105
OPA2Vec	**0.599 ± 0.045**	0.340 ± 0.062	0.388 ± 0.057	0.504 ± 0.105
DL2Vec	**0.709 ± 0.042**	0.434 ± 0.065	0.428 ± 0.058	**0.603 ± 0.103**
OWL2Vec*	**0.675 ± 0.043**	0.426 ± 0.065	0.440 ± 0.060	0.570 ± 0.104

Table S11. Prediction of gene–disease association for diseases mapped to *central nervous system disease* (DOID:331) - Mouse dataset

Method	mouse	fish	fly	yeast
Resnik's	**0.936 ± 0.044**	**0.700 ± 0.115**	**0.751 ± 0.118**	0.427 ± 0.206
OPA2Vec	**0.698 ± 0.082**	0.310 ± 0.116	0.379 ± 0.128	0.473 ± 0.208
DL2Vec	**0.889 ± 0.056**	0.386 ± 0.122	0.516 ± 0.132	0.439 ± 0.207
OWL2Vec*	**0.827 ± 0.067**	0.447 ± 0.124	0.498 ± 0.143	0.397 ± 0.204

Table S12. Prediction of gene–disease association for diseases mapped to *musculoskeletal system disease* (DOID:17) - Human dataset

Method	mouse	fish	fly	yeast
Resnik's	**0.854 ± 0.032**	**0.620 ± 0.053**	**0.593 ± 0.063**	0.533 ± 0.140
OPA2Vec	**0.683 ± 0.042**	0.353 ± 0.052	0.329 ± 0.057	0.444 ± 0.139
DL2Vec	**0.836 ± 0.033**	0.482 ± 0.054	0.390 ± 0.059	0.555 ± 0.139
OWL2Vec*	**0.789 ± 0.037**	0.450 ± 0.054	0.447 ± 0.061	0.427 ± 0.139

Table S13. Prediction of gene–disease association for diseases mapped to *musculoskeletal system disease* (DOID:17) - Mouse dataset

Method	mouse	fish	fly	yeast
Resnik's	**0.964 ± 0.026**	**0.609 ± 0.09**	0.560 ± 0.163	0.589 ± 0.246
OPA2Vec	**0.750 ± 0.061**	0.321 ± 0.086	0.308 ± 0.128	0.524 ± 0.250
DL2Vec	**0.934 ± 0.035**	0.484 ± 0.092	0.353 ± 0.133	0.437 ± 0.248
OWL2Vec*	**0.872 ± 0.047**	0.389 ± 0.090	0.488 ± 0.141	0.564 ± 0.248

Table S14. Prediction of gene–disease association for diseases mapped to *sensory system disease* (DOID:0050155) - Human dataset

Method	mouse	fish	fly	yeast
Resnik's	**0.870** ± **0.035**	0.555 ± 0.067	**0.601** ± **0.076**	0.601 ± 0.188
OPA2Vec	**0.700** ± **0.047**	0.358 ± 0.065	0.301 ± 0.066	0.404 ± 0.189
DL2Vec	**0.825** ± **0.039**	0.437 ± 0.067	0.404 ± 0.070	0.439 ± 0.191
OWL2Vec*	**0.763** ± **0.044**	0.443 ± 0.067	0.364 ± 0.070	0.523 ± 0.192

Table S15. Prediction of gene–disease association for diseases mapped to *sensory system disease* (DOID:0050155) - Mouse dataset

Method	mouse	fish	fly	yeast
Resnik's	**0.972** ± **0.028**	0.585 ± 0.125	0.481 ± 0.208	0.532 ± 0.333
OPA2Vec	**0.805** ± **0.066**	0.375 ± 0.123	0.316 ± 0.170	0.313 ± 0.309
DL2Vec	**0.922** ± **0.045**	0.470 ± 0.127	0.366 ± 0.176	0.516 ± 0.333
OWL2Vec*	**0.851** ± **0.0600**	0.394 ± 0.124	0.359 ± 0.178	0.452 ± 0.332

Table S16. Prediction of gene–disease association for diseases mapped to *brain disease* (DOID:936) - Human dataset

Method	mouse	fish	fly	yeast
Resnik's	**0.788** ± **0.047**	**0.615** ± **0.083**	**0.627** ± **0.077**	0.427 ± 0.139
OPA2Vec	**0.593** ± **0.056**	0.344 ± 0.081	0.386 ± 0.073	0.546 ± 0.139
DL2Vec	**0.703** ± **0.052**	0.471 ± 0.085	0.418 ± 0.074	0.615 ± 0.136
OWL2Vec*	**0.668** ± **0.054**	0.433 ± 0.084	0.464 ± 0.076	0.564 ± 0.139

Table S17. Prediction of gene–disease association for diseases mapped to *brain disease* (DOID:936) - Mouse dataset

Method	mouse	fish	fly	yeast
Resnik's	**0.934** ± **0.057**	**0.697** ± **0.158**	**0.696** ± **0.180**	0.545 ± 0.332
OPA2Vec	**0.716** ± **0.104**	0.351 ± 0.164	0.417 ± 0.186	0.346 ± 0.317
DL2Vec	**0.876** ± **0.076**	0.402 ± 0.168	0.492 ± 0.189	0.347 ± 0.317
OWL2Vec*	**0.858** ± **0.081**	0.521 ± 0.171	0.524 ± 0.196	0.471 ± 0.333

Table S18. Prediction of gene–disease association for diseases mapped to *eye disease* (DOID:5614) - Human dataset

Method	mouse	fish	fly	yeast
Resnik's	**0.880** ± **0.040**	0.567 ± 0.078	0.584 ± 0.094	0.542 ± 0.257
OPA2Vec	**0.725** ± **0.055**	0.357 ± 0.076	0.283 ± 0.079	0.437 ± 0.256
DL2Vec	**0.857** ± **0.043**	0.440 ± 0.078	0.407 ± 0.086	0.364 ± 0.248
OWL2Vec*	**0.773** ± **0.052**	0.454 ± 0.078	0.391 ± 0.087	0.483 ± 0.258

Table S19. Prediction of gene–disease association for diseases mapped to *eye disease* (DOID:5614) - Mouse dataset

Method	mouse	fish	fly	yeast
Resnik's	**0.971** ± **0.033**	0.590 ± 0.142	0.389 ± 0.244	0.525 ± 0.378
OPA2Vec	**0.839** ± **0.071**	0.371 ± 0.139	0.306 ± 0.201	0.315 ± 0.351
DL2Vec	**0.940** ± **0.046**	0.455 ± 0.144	0.327 ± 0.205	0.429 ± 0.374
OWL2Vec*	**0.856** ± **0.068**	0.411 ± 0.142	0.355 ± 0.209	0.505 ± 0.378

Table S20. Prediction of gene–disease association for diseases mapped to *connective tissue disease* (DOID:65) - Human dataset

Method	mouse	fish	fly	yeast
Resnik's	**0.888** ± **0.04**	**0.623** ± **0.077**	**0.618** ± **0.090**	0.610 ± 0.271
OPA2Vec	**0.662** ± **0.06**	0.366 ± 0.077	0.256 ± 0.077	0.433 ± 0.275
DL2Vec	**0.861** ± **0.044**	0.505 ± 0.08	0.388 ± 0.085	0.665 ± 0.262
OWL2Vec*	**0.800** ± **0.050**	0.421 ± 0.079	0.405 ± 0.088	0.523 ± 0.277

Table S21. Prediction of gene–disease association for diseases mapped to *connective tissue disease* (DOID:65) - Mouse dataset

Method	mouse	fish	fly	yeast
Resnik's	**0.963** ± **0.034**	0.599 ± 0.115	0.542 ± 0.257	0.726 ± 0.282
OPA2Vec	**0.716** ± **0.081**	0.363 ± 0.113	0.305 ± 0.181	0.457 ± 0.315
DL2Vec	**0.925** ± **0.047**	0.488 ± 0.117	0.341 ± 0.186	0.366 ± 0.305
OWL2Vec*	**0.873** ± **0.060**	0.379 ± 0.114	0.487 ± 0.196	0.565 ± 0.314

Table S22. Prediction of gene–disease association for diseases mapped to *bone disease* (DOID:0080001) - Human dataset

Method	mouse	fish	fly	yeast
Resnik's	**0.888** ± **0.043**	**0.623** ± **0.082**	**0.606** ± **0.098**	0.661 ± 0.285
OPA2Vec	**0.656** ± **0.065**	0.369 ± 0.081	0.230 ± 0.081	0.426 ± 0.298
DL2Vec	**0.854** ± **0.048**	0.496 ± 0.084	0.381 ± 0.093	0.629 ± 0.291
OWL2Vec*	**0.799** ± **0.055**	0.428 ± 0.083	0.391 ± 0.095	0.500 ± 0.302

Table S23. Prediction of gene–disease association for diseases mapped to *bone disease* (DOID:0080001) - Mouse dataset

Method	mouse	fish	fly	yeast
Resnik's	**0.969** ± **0.033**	0.604 ± 0.121	0.544 ± 0.276	0.687 ± 0.351
OPA2Vec	**0.719** ± **0.087**	0.370 ± 0.120	0.248 ± 0.176	0.513 ± 0.378
DL2Vec	**0.932** ± **0.049**	0.513 ± 0.124	0.335 ± 0.193	0.390 ± 0.369
OWL2Vec*	**0.867** ± **0.065**	0.376 ± 0.120	0.497 ± 0.204	0.624 ± 0.366

Table S24. Prediction of gene–disease association for diseases mapped to *neurodegenerative disease* (DOID:1289) - Human dataset

Method	mouse	fish	fly	yeast
Resnik's	**0.753** ± **0.063**	**0.665** ± **0.100**	**0.656** ± **0.088**	0.549 ± 0.154
OPA2Vec	**0.604** ± **0.072**	0.367 ± 0.102	0.382 ± 0.086	0.454 ± 0.154
DL2Vec	**0.721** ± **0.066**	0.384 ± 0.103	0.486 ± 0.089	0.585 ± 0.152
OWL2Vec*	**0.680** ± **0.069**	0.434 ± 0.104	0.394 ± 0.089	0.627 ± 0.149

Table S25. Prediction of gene–disease association for diseases mapped to *neurodegenerative disease* (DOID:1289) - Mouse dataset

Method	mouse	fish	fly	yeast
Resnik's	**0.942** ± **0.063**	**0.733** ± **0.167**	**0.802** ± **0.159**	0.342 ± 0.254
OPA2Vec	**0.677** ± **0.126**	0.288 ± 0.171	0.424 ± 0.190	0.568 ± 0.265
DL2Vec	**0.905** ± **0.079**	0.382 ± 0.184	0.586 ± 0.190	0.455 ± 0.266
OWL2Vec*	**0.802** ± **0.108**	0.381 ± 0.184	0.498 ± 0.213	0.414 ± 0.263

Table S26. Prediction of gene–disease association for diseases mapped to *immune system disease* (DOID:2914) - Human dataset

Method	mouse	fish	fly	yeast
Resnik's	**0.884** ± **0.049**	0.513 ± 0.126	0.621 ± 0.125	0.486 ± 0.316
OPA2Vec	**0.702** ± **0.070**	0.366 ± 0.121	0.228 ± 0.104	0.501 ± 0.316
DL2Vec	**0.850** ± **0.054**	0.499 ± 0.126	0.437 ± 0.123	0.476 ± 0.316
OWL2Vec*	**0.749** ± **0.066**	0.395 ± 0.123	0.417 ± 0.131	0.474 ± 0.316

Table S27. Prediction of gene–disease association for diseases mapped to *immune system disease* (DOID:2914) - Mouse dataset

Method	mouse	fish	fly	yeast
Resnik's	**0.927** ± **0.064**	0.622 ± 0.177	0.523 ± 0.378	0.609 ± 0.399
OPA2Vec	**0.643** ± **0.118**	0.393 ± 0.178	0.236 ± 0.300	0.508 ± 0.408
DL2Vec	**0.852** ± **0.087**	0.464 ± 0.182	0.394 ± 0.346	0.488 ± 0.408
OWL2Vec*	**0.798** ± **0.099**	0.481 ± 0.182	0.589 ± 0.348	0.647 ± 0.390

Table S28. Prediction of gene–disease association for diseases mapped to *retinal disease* (DOID:5679) - Human dataset

Method	mouse	fish	fly	yeast
Resnik's	**0.880** ± **0.054**	0.546 ± 0.112	0.560 ± 0.130	0.508 ± 0.316
OPA2Vec	**0.772** ± **0.070**	0.412 ± 0.111	0.336 ± 0.113	0.524 ± 0.316
DL2Vec	**0.867** ± **0.057**	0.487 ± 0.112	0.419 ± 0.118	0.395 ± 0.309
OWL2Vec*	**0.815** ± **0.065**	0.406 ± 0.111	0.413 ± 0.120	0.590 ± 0.311

Table S29. Prediction of gene–disease association for diseases mapped to *retinal disease* (DOID:5679) - Mouse dataset

Method	mouse	fish	fly	yeast
Resnik's	**0.984** ± **0.033**	0.506 ± 0.208	0.373 ± 0.306	0.438 ± 0.573
OPA2Vec	**0.877** ± **0.085**	0.411 ± 0.205	0.324 ± 0.250	0.539 ± 0.576
DL2Vec	**0.954** ± **0.054**	0.470 ± 0.208	0.362 ± 0.257	0.519 ± 0.577
OWL2Vec*	**0.912** ± **0.073**	0.358 ± 0.200	0.415 ± 0.263	0.689 ± 0.534

Table S30. Prediction of gene–disease association for diseases mapped to *bone development disease* (DOID:0080006) - Human dataset

Method	mouse	fish	fly	yeast
Resnik's	**0.880** ± **0.053**	**0.624** ± **0.093**	0.597 ± 0.109	0.625 ± 0.323
OPA2Vec	**0.653** ± **0.077**	0.378 ± 0.093	0.234 ± 0.090	0.468 ± 0.333
DL2Vec	**0.857** ± **0.057**	0.487 ± 0.096	0.389 ± 0.104	0.723 ± 0.299
OWL2Vec*	**0.804** ± **0.064**	0.445 ± 0.096	0.376 ± 0.106	0.583 ± 0.329

Table S31. Prediction of gene–disease association for diseases mapped to *bone development disease* (DOID:0080006) - Mouse dataset

Method	mouse	fish	fly	yeast
Resnik's	**0.966** ± **0.042**	**0.642** ± **0.14**	0.512 ± 0.289	0.694 ± 0.376
OPA2Vec	**0.743** ± **0.101**	0.359 ± 0.140	0.214 ± 0.188	0.491 ± 0.408
DL2Vec	**0.944** ± **0.053**	0.535 ± 0.146	0.308 ± 0.212	0.298 ± 0.373
OWL2Vec*	**0.877** ± **0.076**	0.339 ± 0.138	0.442 ± 0.228	0.572 ± 0.404

Table S32. Prediction of gene–disease association for diseases mapped to *hematopoietic system disease* (`DOID:74`) - Human dataset

Method	mouse	fish	fly	yeast
Resnik's	**0.838 ± 0.057**	0.573 ± 0.094	**0.733 ± 0.098**	0.451 ± 0.141
OPA2Vec	**0.701 ± 0.071**	0.368 ± 0.092	0.264 ± 0.092	0.536 ± 0.141
DL2Vec	**0.855 ± 0.055**	**0.625 ± 0.092**	0.462 ± 0.104	0.542 ± 0.141
OWL2Vec*	**0.764 ± 0.066**	0.428 ± 0.094	0.379 ± 0.103	0.480 ± 0.141

Table S33. Prediction of gene–disease association for diseases mapped to *hematopoietic system disease* (`DOID:74`) - Mouse dataset

Method	mouse	fish	fly	yeast
Resnik's	**0.932 ± 0.057**	0.582 ± 0.150	0.644 ± 0.220	0.745 ± 0.436
OPA2Vec	**0.706 ± 0.104**	0.369 ± 0.147	0.241 ± 0.187	0.124 ± 0.329
DL2Vec	**0.916 ± 0.063**	0.528 ± 0.152	0.397 ± 0.214	0.365 ± 0.481
OWL2Vec*	**0.790 ± 0.093**	0.401 ± 0.149	0.409 ± 0.226	0.242 ± 0.428

Table S34. Prediction of gene–disease association for diseases mapped to *primary immunodeficiency disease* (`DOID:612`) - Human dataset

Method	mouse	fish	fly	yeast
Resnik's	**0.884 ± 0.051**	0.481 ± 0.134	0.598 ± 0.137	0.486 ± 0.316
OPA2Vec	**0.706 ± 0.073**	0.357 ± 0.128	0.247 ± 0.115	0.501 ± 0.316
DL2Vec	**0.851 ± 0.057**	0.486 ± 0.134	0.428 ± 0.132	0.476 ± 0.316
OWL2Vec*	**0.741 ± 0.070**	0.383 ± 0.130	0.400 ± 0.141	0.474 ± 0.316

Table S35. Prediction of gene–disease association for diseases mapped to *primary immunodeficiency disease* (`DOID:612`) - Mouse dataset

Method	mouse	fish	fly	yeast
Resnik's	**0.923 ± 0.069**	0.601 ± 0.188	0.523 ± 0.378	0.609 ± 0.399
OPA2Vec	**0.627 ± 0.126**	0.371 ± 0.186	0.236 ± 0.300	0.508 ± 0.408
DL2Vec	**0.847 ± 0.094**	0.447 ± 0.191	0.394 ± 0.346	0.488 ± 0.408
OWL2Vec*	**0.787 ± 0.107**	0.453 ± 0.192	0.589 ± 0.348	0.647 ± 0.390

Table S36. Prediction of gene–disease association for diseases mapped to *disease of mental health* (`DOID:150`) - Human dataset

Method	mouse	fish	fly	yeast
Resnik's	**0.732 ± 0.078**	0.564 ± 0.110	0.550 ± 0.104	0.620 ± 0.235
OPA2Vec	0.537 ± 0.087	0.443 ± 0.110	0.315 ± 0.092	0.487 ± 0.242
DL2Vec	**0.625 ± 0.085**	0.477 ± 0.111	0.379 ± 0.096	0.530 ± 0.242
OWL2Vec*	**0.618 ± 0.085**	0.548 ± 0.111	0.454 ± 0.101	0.482 ± 0.242

Table S37. Prediction of gene–disease association for diseases mapped to *disease of mental health* (`DOID:150`) - Mouse dataset

Method	mouse	fish	fly	yeast
Resnik's	**0.863 ± 0.082**	0.575 ± 0.165	**0.727 ± 0.190**	0.587 ± 0.372
OPA2Vec	0.583 ± 0.118	0.446 ± 0.166	0.411 ± 0.201	0.293 ± 0.344
DL2Vec	**0.677 ± 0.112**	0.363 ± 0.160	0.333 ± 0.192	0.509 ± 0.378
OWL2Vec*	**0.656 ± 0.114**	0.513 ± 0.167	0.494 ± 0.213	0.452 ± 0.376

Disease Models & Mechanisms • Supplementary information

Table S38. Prediction of gene–disease association for diseases mapped to *mitochondrial metabolism disease* (DOID:700) - Human dataset

Method	mouse	fish	fly	yeast
Resnik's	0.510 ± 0.099	0.576 ± 0.233	0.528 ± 0.135	0.423 ± 0.114
OPA2Vec	**0.612 ± 0.097**	0.498 ± 0.236	0.461 ± 0.119	0.551 ± 0.115
DL2Vec	**0.659 ± 0.094**	0.478 ± 0.235	0.514 ± 0.119	0.596 ± 0.113
OWL2Vec*	**0.666 ± 0.094**	0.478 ± 0.235	0.523 ± 0.125	0.493 ± 0.115

Table S39. Prediction of gene–disease association for diseases mapped to *mitochondrial metabolism disease* (DOID:700) - Mouse dataset

Method	mouse	fish	fly	yeast
Resnik's	**0.832 ± 0.207**	0.418 ± 0.493	0.540 ± 0.377	0.248 ± 0.288
OPA2Vec	0.729 ± 0.247	0.355 ± 0.479	0.443 ± 0.351	0.713 ± 0.302
DL2Vec	**0.905 ± 0.163**	0.099 ± 0.299	0.400 ± 0.346	0.641 ± 0.320
OWL2Vec*	**0.891 ± 0.173**	0.610 ± 0.488	0.832 ± 0.374	0.417 ± 0.329

Table S40. Prediction of gene–disease association for diseases mapped to *muscular disease* (DOID:0080000) - Human dataset

Method	mouse	fish	fly	yeast
Resnik's	**0.810 ± 0.065**	**0.645 ± 0.085**	0.544 ± 0.111	0.569 ± 0.202
OPA2Vec	**0.711 ± 0.075**	0.362 ± 0.085	0.377 ± 0.105	0.398 ± 0.200
DL2Vec	**0.805 ± 0.066**	0.495 ± 0.089	0.403 ± 0.106	0.511 ± 0.204
OWL2Vec*	**0.793 ± 0.067**	0.466 ± 0.089	0.463 ± 0.110	0.374 ± 0.198

Table S41. Prediction of gene–disease association for diseases mapped to *muscular disease* (DOID:0080000) - Mouse dataset

Method	mouse	fish	fly	yeast
Resnik's	**0.970 ± 0.050**	0.668 ± 0.175	0.480 ± 0.301	0.456 ± 0.704
OPA2Vec	**0.797 ± 0.119**	0.263 ± 0.163	0.335 ± 0.262	0.476 ± 0.706
DL2Vec	**0.934 ± 0.073**	0.536 ± 0.185	0.384 ± 0.270	0.429 ± 0.700
OWL2Vec*	**0.887 ± 0.093**	0.343 ± 0.176	0.526 ± 0.301	0.317 ± 0.658

Table S42. Prediction of gene–disease association for diseases mapped to *integumentary system disease* (DOID:16) - Human dataset

Method	mouse	fish	fly	yeast
Resnik's	**0.840 ± 0.061**	0.595 ± 0.130	**0.617 ± 0.116**	0.548 ± 0.182
OPA2Vec	**0.712 ± 0.076**	0.483 ± 0.132	0.279 ± 0.102	0.495 ± 0.183
DL2Vec	**0.849 ± 0.060**	0.550 ± 0.132	0.356 ± 0.108	0.557 ± 0.181
OWL2Vec*	**0.728 ± 0.074**	0.525 ± 0.132	0.389 ± 0.110	0.420 ± 0.180

Table S43. Prediction of gene–disease association for diseases mapped to *integumentary system disease* (DOID:16) - Mouse dataset

Method	mouse	fish	fly	yeast
Resnik's	**0.963 ± 0.054**	0.638 ± 0.240	0.573 ± 0.330	0.537 ± 0.499
OPA2Vec	**0.797 ± 0.115**	0.593 ± 0.246	0.351 ± 0.288	0.578 ± 0.494
DL2Vec	**0.935 ± 0.070**	0.550 ± 0.249	0.388 ± 0.294	0.517 ± 0.500
OWL2Vec*	**0.857 ± 0.100**	0.674 ± 0.234	0.374 ± 0.292	0.383 ± 0.486

Table S44. Prediction of gene–disease association for diseases mapped to *cardiovascular system disease* (DOID:1287) - Human dataset

Method	mouse	fish	fly	yeast
Resnik's	**0.891** ± **0.050**	**0.664** ± **0.092**	**0.617** ± **0.101**	0.510 ± 0.447
OPA2Vec	**0.696** ± **0.074**	0.329 ± 0.143	0.267 ± 0.088	0.362 ± 0.430
DL2Vec	**0.861** ± **0.056**	0.329 ± 0.091	0.408 ± 0.098	0.391 ± 0.436
OWL2Vec*	**0.751** ± **0.070**	0.413 ± 0.096	0.422 ± 0.101	0.702 ± 0.409

Table S45. Prediction of gene–disease association for diseases mapped to *cardiovascular system disease* (DOID:1287) - Mouse dataset

Method	mouse	fish	fly	yeast
Resnik's	**0.949** ± **0.051**	**0.690** ± **0.141**	0.693 ± 0.224	0.776 ± 0.295
OPA2Vec	**0.761** ± **0.099**	0.329 ± 0.143	0.310 ± 0.218	0.293 ± 0.322
DL2Vec	**0.927** ± **0.060**	0.347 ± 0.145	0.305 ± 0.217	0.255 ± 0.308
OWL2Vec*	**0.852** ± **0.083**	0.465 ± 0.152	0.272 ± 0.216	0.501 ± 0.354

Table S46. Prediction of gene–disease association for diseases mapped to *muscle tissue disease* (DOID:66) - Human dataset

Method	mouse	fish	fly	yeast
Resnik's	**0.820** ± **0.065**	**0.653** ± **0.086**	0.540 ± 0.114	0.556 ± 0.207
OPA2Vec	**0.724** ± **0.076**	0.369 ± 0.087	0.387 ± 0.108	0.393 ± 0.204
DL2Vec	**0.822** ± **0.065**	0.491 ± 0.091	0.401 ± 0.109	0.530 ± 0.208
OWL2Vec*	**0.810** ± **0.067**	0.471 ± 0.090	0.469 ± 0.113	0.374 ± 0.202

Table S47. Prediction of gene–disease association for diseases mapped to *muscle tissue disease* (DOID:66) - Mouse dataset

Method	mouse	fish	fly	yeast
Resnik's	**0.971** ± **0.050**	**0.682** ± **0.179**	0.480 ± 0.301	0.456 ± 0.704
OPA2Vec	**0.814** ± **0.116**	0.275 ± 0.172	0.335 ± 0.262	0.476 ± 0.706
DL2Vec	**0.949** ± **0.065**	0.515 ± 0.192	0.384 ± 0.270	0.429 ± 0.700
OWL2Vec*	**0.890** ± **0.093**	0.352 ± 0.184	0.526 ± 0.301	0.317 ± 0.658

Table S48. Prediction of gene–disease association for diseases mapped to *peripheral nervous system disease* (DOID:574) - Human dataset

Method	mouse	fish	fly	yeast
Resnik's	**0.799** ± **0.068**	0.566 ± 0.112	**0.655** ± **0.111**	0.404 ± 0.205
OPA2Vec	**0.663** ± **0.080**	0.308 ± 0.105	0.346 ± 0.103	0.569 ± 0.207
DL2Vec	**0.802** ± **0.067**	0.401 ± 0.111	0.369 ± 0.104	0.543 ± 0.208
OWL2Vec*	**0.746** ± **0.074**	0.500 ± 0.113	0.456 ± 0.110	0.448 ± 0.207

Table S49. Prediction of gene–disease association for diseases mapped to *peripheral nervous system disease* (DOID:574) - Human dataset

Method	mouse	fish	fly	yeast
Resnik's	**0.799** ± **0.068**	0.566 ± 0.112	**0.655** ± **0.111**	0.404 ± 0.205
OPA2Vec	**0.663** ± **0.080**	0.308 ± 0.105	0.346 ± 0.103	0.569 ± 0.207
DL2Vec	**0.802** ± **0.067**	0.401 ± 0.111	0.369 ± 0.104	0.543 ± 0.208
OWL2Vec*	**0.746** ± **0.074**	0.500 ± 0.113	0.456 ± 0.110	0.448 ± 0.207

Table S50. Prediction of gene–disease association for diseases mapped to *developmental disorder of mental health* (DOID:0060037) - Human dataset

Method	mouse	fish	fly	yeast
Resnik's	**0.719** $\pm$ **0.087**	0.563 $\pm$ 0.121	0.537 $\pm$ 0.111	0.665 $\pm$ 0.252
OPA2Vec	0.528 $\pm$ 0.097	0.451 $\pm$ 0.122	0.293 $\pm$ 0.096	0.476 $\pm$ 0.267
DL2Vec	**0.606** $\pm$ **0.095**	0.501 $\pm$ 0.122	0.380 $\pm$ 0.102	0.557 $\pm$ 0.266
OWL2Vec*	**0.627** $\pm$ **0.094**	0.561 $\pm$ 0.121	0.451 $\pm$ 0.107	0.508 $\pm$ 0.267

Table S51. Prediction of gene–disease association for diseases mapped to *developmental disorder of mental health* (DOID:0060037) - Mouse dataset

Method	mouse	fish	fly	yeast
Resnik's	**0.827** $\pm$ **0.178**	0.645 $\pm$ 0.428	0.561 $\pm$ 0.444	0.532 $\pm$ 0.499
OPA2Vec	0.576 $\pm$ 0.233	0.286 $\pm$ 0.404	0.166 $\pm$ 0.333	0.199 $\pm$ 0.399
DL2Vec	0.689 $\pm$ 0.218	0.247 $\pm$ 0.386	0.291 $\pm$ 0.406	0.567 $\pm$ 0.496
OWL2Vec*	0.682 $\pm$ 0.220	0.467 $\pm$ 0.446	0.383 $\pm$ 0.435	0.534 $\pm$ 0.499

Supplementary Materials and Methods

In order to test the hypothesis that *Drosophila* phenotype (DP) classes are not well connected with specific human phenotype classes, we define MIS-HP. MIS-HP is the most informative superclass from human phenotypes to a certain class. Let C be a *Drosophila* phenotype class, we define MIS-HP for this class as shown in equation 1. Then we calculate the absolute difference between the information content of DP and its MIS-HP, we will use ($sum\Delta_G$) to refer to some of this difference for all the annotations of a gene. Let G_i be the ith annotation for a gene, we calculate $sum\Delta_G$ as shown in equation 2.

$$MIS-HP(C) = \max_{P \in Superclasses(C) \cap HP} IC(P) \quad (1)$$

$$sum\Delta_G = \sum_i \Big| IC(G_i) - IC(MIS-HP(G_i)) \Big| \quad (2)$$

$$avg\Delta_G = \frac{\sum_i \Big| IC(G_i) - IC(MIS-HP(G_i)) \Big|}{n} \quad (3)$$

To visualize the difference between the associated genes and the non-associated genes, we calculate $avg\Delta_G$ as the average of the absolute difference between DP and its $MIS-HP$. Let n be the number of annotations a gene have, equation 3 shows how we calculate $avg\Delta_G$. Figure S2 shows the density histogram for the $avg\Delta_G$ for disease-associated genes and non-disease associated genes.

We compare the list of $sum\Delta_G$ for the disease-associated genes and the non-disease-associated genes. This comparison is done using a t-test and the result shows that $sum\Delta_G$ for the disease-association genes is significantly higher than the non-disease-associations p-value ($p = 4 \times 10^{-61}$), and mean 44.5 for disease-associated genes and mean 13.9 for non-disease-associated genes.

Disease Models & Mechanisms • Supplementary information
